# Retroperitoneal Tumor, a Primary Cavernous Hemangioma: A Case Report

**DOI:** 10.7759/cureus.43442

**Published:** 2023-08-13

**Authors:** Nasser AlBishi, Mohammed Alwhabi, Mohammed Abdelatif M Elhassan

**Affiliations:** 1 Pathology and Laboratory Medicine, King Fahad Medical City, Riyadh, SAU; 2 Anatomical Pathology, Prince Sultan Military Medical City, Riyadh, SAU; 3 Anatomical Pathology, King Fahad Medical City, Riyadh, SAU

**Keywords:** hemangioma, hemangioma cavernous therapy, retroperitoneal sarcomas, retroperitoneal hemangioma, cavernous hemangioma

## Abstract

The diagnosis of primary retroperitoneal cavernous hemangiomas is extremely rare in clinical practice. Only a few cases have been reported. Due to the lack of specific radiological features, their diagnosis is uncommon. They are usually found incidentally or after symptoms as a consequence of complications. Adult retroperitoneal cavernous hemangiomas are extremely rare. This is a report of a rare case of a primary retroperitoneal cavernous hemangioma in a 45‐year‐old male patient discovered after acute appendicitis. A histopathological examination is conducted following total surgical resection to confirm the diagnosis.

## Introduction

Primary retroperitoneal hemangiomas are extremely rare. Between 1950 and 2022, fewer than 30 cases of adult retroperitoneal hemangiomas have been reported in the literature, and only few of these 30 cases are primary with no organ or vessel connections [1]. Renal hemangiomas are the most common retroperitoneal hemangiomas in adults. The adrenal glands and pancreas can also be affected by hemangiomas [2,3]. In this paper, we report an adult case of a primary retroperitoneal hemangioma with extensive necrosis and hemorrhage that suggested sarcoma or gastrointestinal stromal tumor (GIST) radiologically.

## Case presentation

The patient was a 43-year-old, male who presented to a primary center with acute abdominal pain and was admitted for an open appendectomy. During the surgery, they found a retroperitoneal mass and described it as a mass abutting the left kidney. After that, the patient was referred to our center.

**Figure 1:**
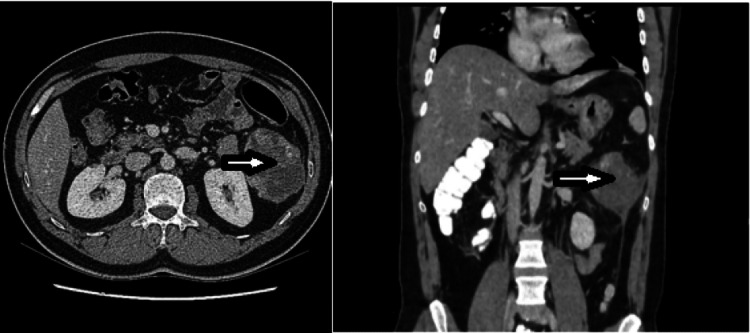
CT images CT abdomen and pelvis show a non-enhancing mass cystic and solid components abutting the kidney and a hypo-dense center (arrow).

CT abdomen and pelvis was performed in the portal venous phase and showed a 7.9 x 7.3 x 7.2 cm mass abutting the kidney with involvement of the left peritoneal reflection and abutting the adjacent descending colon, a heterogeneous enhancement that suggests cystic and solid components. It showed a hypo-dense center that could be necrosis. No calcification could be appreciated. No definite invasion of the spleen or bowel. Radiology suggests that the mass is highly suspicious for sarcoma (Figure 1).

**Figure 2:**
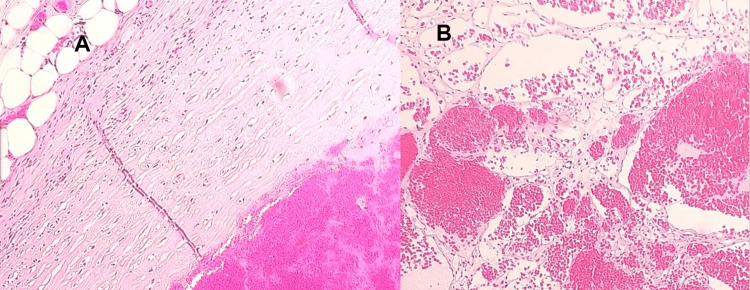
10x hematoxylin and eosin (H&E) A. Tumor shows a thick fibrous pseudocapsule. B. Cavernous vascular proliferation, variable-size venular/capillary structures.

**Figure 3:**
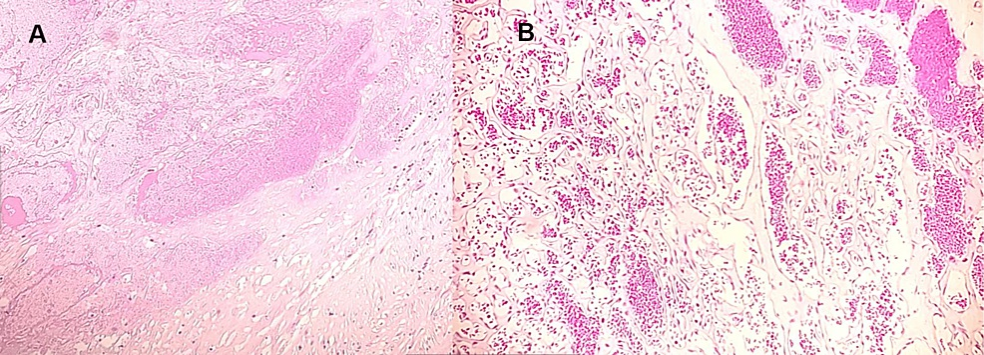
10x hematoxylin and eosin (H&E) A. Central necrosis and hyalinization. B. Areas of resembling Intravascular papillary endothelial hyperplasia.

After laparoscopic retroperitoneal excision, pathologic examination showed a 7 x 5 x 4 cm encapsulated nodular mass with extensive central necrosis and hemorrhage lesion. The section shows a well-defined lesion with a fibrous pseudo-capsule, composed of cavernous vascular proliferation and variable-size venular/capillary structures (Figure 2). The central part of the lesion shows extensive infarction and hyalinization. The peripheral areas of the necrotic tissue show focal endothelial papillary hyperplasia within vascular spaces lined by a single layer of plump endothelial cells resembling intravascular papillary endothelial hyperplasia. No atypia, mitosis, or apparent infiltrative growth was identified (Figure 3). The final diagnosis was a benign vasoformative lesion consistent with cavernous hemangioma. The tumor board considered no role for chemotherapy or radiotherapy (XRT) and further management other than future follow-up.

## Discussion

Among all tumors, retroperitoneal neoplasms account for only 0.1-0.2%. The most common type of retroperitoneal neoplasm is mesodermal, neurogenic, or lymphatic in origin [4]. A retroperitoneal hemangioma is rare and is almost always of the cavernous type [5]. There are several common locations where retroperitoneal cavernous hemangiomas can occur, including the peripancreatic area, perirenal area, and periureteric region. Genitourinary cavernous hemangiomas most commonly occur in the kidney and bladder and can involve some rare locations, such as the ilium [6,7]. Cavernous hemangiomas in the abdomen or retroperitoneum do not cause any specific symptoms. As symptoms of renal cavernous hemangiomas, patients may experience flank pain, hematuria, anemia, thrombocytopenia, renal vein thrombosis (rarely), or even life-threatening bleeding [8].

Between 1950 and 2022, fewer than 30 cases of adult retroperitoneal hemangiomas have been reported in the literature, including 19 cases in Japan [1]. Hemangiomas arising from the infundibulopelvic vessels are rarely found; as such, only two cases have been reported [9]. Pancreatic cavernous hemangiomas are cystic tumors that usually lack larger supply vessels, resulting in a lack of arterial enhancement on radiographs and hence wrong diagnoses [10]. In retroperitoneal hemangiomas that are asymptomatic, no further treatment is needed; however, if the tumor grows, it oppresses adjacent organs or develops symptoms that are not specific [10,11].

## Conclusions

Retroperitoneal cavernous hemangiomas are a rare type of retroperitoneal tumor. A primary retroperitoneal cavernous hemangioma is presented in this case report. The tumor was abutting the colon and kidney, but it was not connected to any specific feeding artery, so it was diagnosed as a primary retroperitoneal cavernous hemangioma. While a retroperitoneal cavernous hemangioma is a benign lesion, it is difficult to diagnose preoperatively clinically, and more often than not, sarcoma is diagnosed. Accordingly, surgical resection is a curative treatment.
